# Exploring the relationship between static and dynamic balance performance through the same center-of-pressure parameters

**DOI:** 10.1186/s13102-025-01251-x

**Published:** 2025-07-30

**Authors:** A. Rizzato, A. Paoli, Giuseppe Marcolin

**Affiliations:** Department of Biomedical Sciences, University of Padova, Via Marzolo, 3, Padua, 35131 Italy

**Keywords:** Unstable board, Center of pressure, Isometric strength, Maximal voluntary contraction

## Abstract

**Background:**

The interpretation of evidence on the relationship between static and dynamic balance is complicated due to the several systems involved in postural control and the heterogeneity of the dynamic balance tasks used. The primary aim of this study was to explore the correlation between static and dynamic balance performance among healthy adults by means of the same center-of-pressure parameters. Given the importance of rapid reactive postural response in dynamic conditions, the secondary aim was to explore the relationship between dynamic balance performance and quadriceps strength and power.

**Methods:**

Thirty-two healthy subjects (18 females; mean ± SD: age = 30.68 ± 13.31 years; body mass = 74.84 ± 15.18 kg; height = 1.75 ± 0.07 m) were assessed in static and dynamic balance conditions through a force plate that allowed computing the center of pressure trajectory. Static balance was assessed during an upright standing test and dynamic balance during an unstable board test. The same center-of-pressure parameters were calculated for both the two balance conditions: the 95th percentile ellipse area (Area95) and center-of-pressure mean velocity (MeanVelocity). The isometric quadriceps strength of the dominant leg was measured at 90 degrees of knee flexion with a load cell in steady and ballistic conditions. The maximal isometric strength and rate of force development were calculated.

**Results:**

Pearson’s correlation showed non-statistically significant correlations between static and dynamic balance performance for both Area95 (R^2^ = 0.10; *p* = 0.07) and MeanVelocity (R^2^ = 0.001; *p* = 0.99). Across all parameters, the stepwise multiple linear regression analysis identified the RFD in the 100–150 ms window as the only determinant factor of the Area95 (*p* < 0.05, adjusted R² = 0.136) and MeanVelocity (*p* < 0.05, adjusted R² = 0.188) in the dynamic balance condition.

**Conclusions:**

This study suggests a lack of correlation between static and dynamic balance performance in healthy adults, indicating that both may need to be considered in balance assessments for a more comprehensive evaluation.

**Supplementary Information:**

The online version contains supplementary material available at 10.1186/s13102-025-01251-x.

## Introduction

Postural control is a complex motor function that relies on interactions among different sensorimotor processes, with primary goals of body stability, orientation, and balance [1]. Movement strategies and muscle synergies must be coordinated to maintain balance and reduce shifts of the center of pressure (CoP) [2]. Originally, literature focused on defining static and dynamic balance. Under static conditions, the base of support provided by the feet remains stationary, while the CoP exhibits minimal displacements in undisturbed environments [3, 4]. In contrast, under dynamic conditions, the base of support can move, or external mechanical perturbations (i.e., expected or unexpected) can occur, requiring a higher challenge in keeping the CoP within the base of support [5, 6].

Although force platforms are the most widely used devices in assessing postural function through the CoP trajectory calculation [4], it is surprising to note that no one in the literature investigated the relationship between static and dynamic balance tests considering the same CoP-related parameters. The dynamic balance was mostly evaluated using non-instrumental field tests (e.g., timed figure-eight run, timed-up-and-go, functional reach). Only two studies [7, 8] investigated the relationship between static (i.e., single or double-leg standing) and dynamic balance on a force platform. While some weak correlations were identified, the dynamic test administered by the authors (i.e., single leg landing and stepping task) did not completely fit the definition of dynamic balance [5, 6]. Thus, given that the assessment of previous studies did not meet the definition of dynamic balance test, nor did it use objective parameters based on the CoP trajectory, further research with the same consistent CoP-related parameters seems necessary to deepen the static and dynamic balance relationship.

In addition, in more functional field settings, individuals with greater muscular strength and power exhibited a lower risk of falling and performed better on balance tasks compared to those with lower levels of strength and power [9, 10]. Thus, strength and power appear as important physiological attributes to enhance balance performance. In particular, the role of lower-limb strength—especially quadriceps strength—has gained attention due to its crucial involvement in stabilization and movement during balance tasks [11–13]. The integration of strength assessments, alongside balance tests, helps clarify whether muscular performance underpins balance performance under dynamic conditions. Motor output of the postural control is closely related to lower-limb muscle strength and power and proves to be a determining factor in balance performance [14]. However, most studies investigating the relationship between strength or power and balance were performed in older adults where the impact of neuromuscular aging is not negligible. A systematic review and meta-analysis showed small-sized correlations between measures of balance and lower-extremity muscle strength or power across the lifespan [15]. Theoretically, muscle power should be more important under dynamic conditions than maximal strength, as the speed of force application becomes crucial when the stance or ground is moving [14]. Indeed, rapid force expression (i.e., ballistic contraction) may support a direct association with balance performance in reactive dynamic tasks. Ballistic contractions are characterized by high firing rates, brief contraction times, and high rates of force development [16]. Conversely, steady contractions kept persistent muscular tension and generate a maintained force [16]. In the relationship between strength and balance the latter has been investigated by timing dynamic tasks (e.g., gait, chair rise, timed up and go, stair climbing) of different complexity [17, 18]. Therefore, regarding the relevance of previous investigations on the interplay between static and dynamic balance performance and muscle strength, the purpose of this study was twofold. First, the study aimed to investigate whether a relationship between static and dynamic balance performance could exist in a group of healthy adults. Based on the assumption that balance is task-specific [19–21], we hypothesized that static and dynamic balance performance would not be correlated. Second, the study aimed to examine whether dynamic balance performance is associated with maximal isometric strength and power, particularly under ballistic than steady muscle contractions. Given the importance of rapid postural responses in dynamic balance management, we hypothesized a relationship between dynamic balance performance and strength and power during ballistic contractions.

## Methods

### Subjects

The a priori power analysis calculation (G*Power 3.1.9.2, Heinrich Heine University, Düsseldorf, Germany) showed that a sample size of 30 participants and a correlation ρH1 of 0.6 would provide a statistical Power of 0.9. Thirty-two healthy young subjects (Females = 18; age: 30.68 ± 13.31 years; mass: 74.84 ± 15.18 kg; height: 1.75 ± 0.07 m) volunteered for the study. Before enrollment, all the subjects were screened through a telephone interview, and all were eligible for the study. The subjects were included whether they had self-reported no previous practice with unstable board training. Moreover, the following exclusion criteria were considered: no history of (i) orthopedic injuries in the last 6 months, (ii) neurological diseases, and (iii) sight, hearing, or vestibular disorders. Subjects were informed about the experimental procedures before providing written informed consent to participate in the study.

### Study design

The experimental protocol received approval by the Human Ethical Committee of the Department of Biomedical Sciences of the University of Padova (n° HEC-DSB/05–21; Clinical trial number: not applicable) and adhered to the principles of the Declaration of Helsinki. This cross-sectional study design evaluated bipedal static and dynamic balance performance and maximal quadriceps strength during an isometric leg extension task. Subjects underwent an evaluation session of balance tests in static and dynamic conditions on a 60 cm × 40 cm dynamometric platform (AMTI BP400600, Watertown, MA, USA). Data collection was performed at a sampling frequency of 200 Hz, and the software Balance Clinic 1.4.2 was used to calculate the CoP trajectory. Dynamic balance assessment was performed using an unstable board on the dynamometric platform [22]. Strength tests were performed on a custom-built chair, and maximal isometric performance was assessed through a load cell. Before each testing procedure, researchers guaranteed a familiarization session to explain the scheduled program and guarantee its correct execution.

### Measurements

#### Static balance

During the bipedal static balance test, subjects were instructed to stand upright with their arms at their sides. They were asked to gaze at a thin red line vertically placed on a white wall in front of them at a distance of 80 cm. The foot positioning on the dynamometric platform was standardized using a V-shaped frame, maintaining a 7 cm distance between the heels and a 30° angle between the toes, following the recommendations of the International Society of Posturography [23]. Three trials, each lasting 30 s, were conducted with a 60-second recovery period between each trial.

#### Dynamic balance

Subjects stood on the unstable board during the bipedal dynamic balance test using their preferred stance width. Specifically, the unstable board had only one degree of freedom, allowing rotational movement only in the forward and backward directions. Subjects were instructed to keep the board as parallel to the ground as possible without moving their feet from their original positions. Subjects were asked to gaze at the same target used in the static balance test. Subjects kept their hands on their hips throughout the test to prevent counterbalancing actions. The unstable board (length: 50 cm; width: 50 cm; height: 8.5 cm; radius: 10.95 cm) was placed on the dynamometric platform to calculate the CoP trajectory. It rotated 16 degrees anteriorly and posteriorly. The trial was considered invalid if the subject moved the hands from the hips or made a step out of the board. Three trials, each lasting 30 s, were conducted with 60 s of recovery between each trial.

#### Isometric lower-limb strength

The maximal isometric strength of the dominant quadriceps for both steady (ST) and ballistic (BAL) conditions was assessed. For this measurement, we used a custom-built chair [16, 24] and a load cell (MuscleLab™ 4100e; Ergotest Innovation, Porsgrunn, Norway). The load cell was positioned three centimeters above the lateral malleolus of the dominant leg, perpendicular to the subject’s shank [16]. Subjects were required to sit with their knee at a 90-degree angle. Chest and leg straps were used to prevent additional movements during the strength test. Following a standardized warm-up, which included a 10-minute walk on a treadmill at 5 km/h and 10 repetitions of a half-squat exercise, each subject became familiar with the experimental setup by performing five contractions at a submaximal self-selected intensity for both BAL and ST conditions. After a 5-minute rest, subjects conducted the test without any visual feedback. For each of the two conditions (i.e., BAL and ST), subjects were required to exert the maximal voluntary isometric contraction (MVIC). Three consecutive trials were performed for each condition, with a 40-second recovery period between trials. During each MVIC, the operator provided verbal prompts to the subject [25]. In the BAL condition, the subjects were asked to reach the maximal force as fast as possible. In the ST condition, subjects were allowed to reach the maximal force without any constraints but were required to maintain it for 3 s. A 4-minute rest occurred between BAL and ST conditions.

### Data analysis

#### Static balance and dynamic balance

**Fig. 1:**
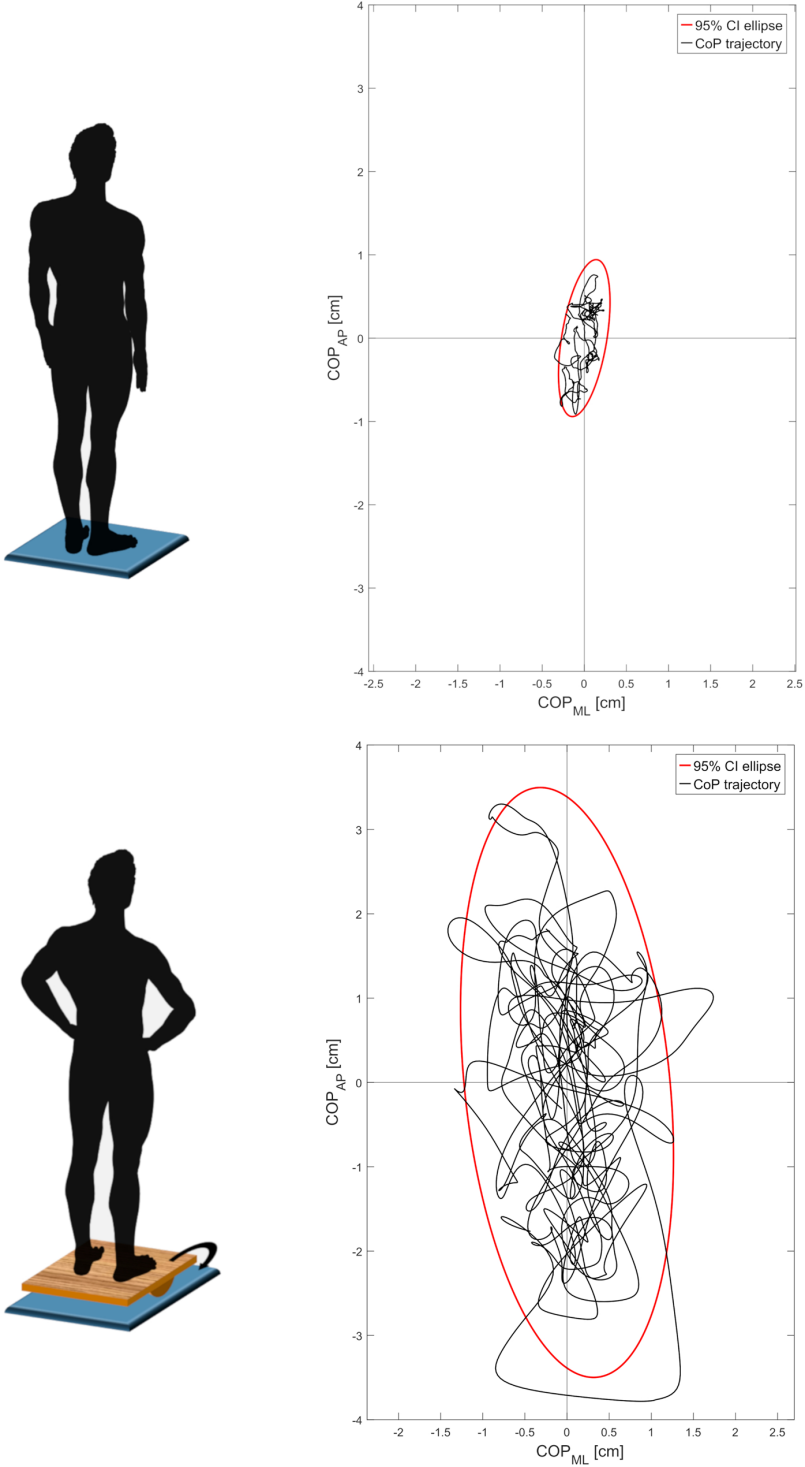
Graphical example of the CoP trajectory during static and dynamic balance conditions, of a representative subject. The 95th percentile ellipse is depicted by the red line

On the CoP trajectory (Fig. 1**)**, the following parameters were calculated: Area95 (i.e., the area of the 95th percentile ellipse measured in cm^2^) and MeanVelocity (the path length per time unit, i.e., the average velocity in cm/s). Area95 serves as a comprehensive measure of postural performance, reflecting the extent of sway or stability [4]. MeanVelocity, on the other hand, is a critical indicator of the efficiency of postural adjustments, with higher velocities typically indicating more adjustments per unit of time, and thus less efficient control [4]. The CoP parameters were averaged among the three trials.

#### Isometric strength and power

The highest lower-limb peak force was considered among the three trials and normalized to body weight in both steady (FMAX_ST_) and ballistic (FMAX_BAL_) conditions. In the BAL trials, the rate of force development (RFD) was calculated as the slope of the force-time curve over the following time windows from the onset of force production: 0–50 ms (RFD_0-50_), 50–100 ms (RFD_50-100_), and 100–150 ms (RFD_100-150_). The onset of contraction was determined when the force signal reached a threshold of 2% of the MVIC values [26, 27], using an automated detection technique.

### Statistical analysis

The Shapiro-Wilk test was used to check the normality of data distribution. Data points that were more than three times the interquartile range (IQR) away from the quartiles were considered outliers and were subsequently removed. The reliability of the CoP-related parameters between the three ST repetitions and between the three BAL repetitions was investigated through the Intraclass Correlation Coefficient (ICC) and the relative 95% confidence interval (95% CI). Specifically, intrasession ICC [3, k] estimated correlations averaged across the k measurements, where in our study, k = 3. ICC coefficients were interpreted as poor (below 0.50), moderate (0.50–0.75), good (0.75–0.90), and excellent (above 0.90) [28]. The mean values, standard deviation (SD), and coefficient of variation (CV) were calculated for all variables. Pearson’s correlation coefficient (r) was used to assess any relationship between the CoP-related measures in static and dynamic balance tests. For the dynamic balance performance, stepwise multiple linear regression analyses were performed with the Area95 and MeanVelocity as dependent variables and RFD parameters and isometric maximal strength (i.e., FMAX_ST_ and FMAX_BAL_) as independent variables. The amount of variance explained by each of the correlations (R^2^) and by the predictive models (adjusted R^2^) were also reported. The strength of the correlations was interpreted [29] as absent to little (< 0.25), fair (0.25–0.49), moderate (0.5–0.74), and very good to excellent (> 0.75). The significance level was set at *p* < 0.05. All analyses were performed using the statistical package JASP (Version 0.19.1).

## Results

All the subjects completed the study. Table 1 shows the mean scores, standard deviations, and coefficient of variations of the CoP-related parameters in the static and dynamic balance tests.

**Table 1:** Summary of results for balance and strength parameters. Data are presented as mean, standard deviation (SD), and coefficient of variation (CV). RFD: rate of force Development. /: not applicable

	**BALANCE**					
	STATIC			DYNAMIC		
	Mean	SD	CV	Mean	SD	CV
Area95 (cm^2^)	1.15	0.66	0.57	20.61	7.79	0.38
Mean Velocity (cm/s)	3.05	0.56	0.18	7.13	1.60	0.22
	**STRENGTH**					
	STEADY			BALLISTIC		
	Mean	SD	CV	Mean	SD	CV
F_max_ (N)	0.79	0.25	0.32	0.65	0.27	0.41
RFD_50 − 100_ (N/s)	/	/	/	3194.40	1944.01	0.61
RFD_100 − 150_ (N/s)	/	/	/	2462.35	1281.48	0.52
RFD_150 − 200_ (N/s)	/	/	/	1534.11	901.04	0.69

**Fig. 2:**
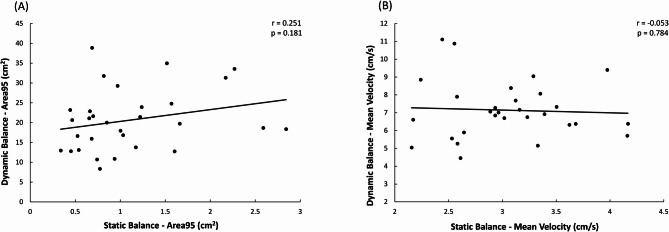
Pearson’s correlations between static and dynamic balance conditions for Area95 (**A**) and MeanVelocity (**B**)

The results of the correlational analysis between static and dynamic balance performance are presented in Fig. 2. For the Area95 parameter (Fig. 2A), Pearson’s correlation showed non-statistically significant correlations between static and dynamic balance performance (R^2^ = 0.06). Similarly, for the MeanVelocity parameter (Fig. 2B), results did not show any significant correlation between the static and dynamic balance performance (R^2^ = 0.002). Results of the intrasession reliability analysis are provided in Table 2. In the dynamic balance performance, the multiple linear regression showed that the model referred to the Area95 explained 13.6% of the variance (adjusted R^2^ = 0.136). The overall F-test yielded an F-statistic of 5.410 (*p* < 0.05), indicating that at least one of the predictors had a significant effect. Indeed, only the RFD_100 − 150_ contributed to the model in determining the Area95 (*p* < 0.05). Similarly, the model for MeanVelocity explained the 18.8% of the variance (adjusted R^2^ = 0.188). The overall F-test resulted in an F-statistic of 7.233 (*p* < 0.05), with RFD_100 − 150_ again being the sole significant predictor for MeanVelocity (*p* < 0.05).

**Table 2:** Average intrasession [3, *k*] intraclass correlation coefficient (ICC) and 95% confidence intervals (95% CI) were calculated for the static and dynamic balance test

		ICC	95% CI
Static Balance	Area95 (cm^2^)	0.880	0.754–0.941
	Mean Velocity (cm/s)	0.992	0.986–0.996
Dynamic Balance	Area95 (cm^2^)	0.436	–0.015–0.706
	Mean Velocity (cm/s)	0.918	0.852–0.957

## Discussion

### Relationship between static and dynamic balance

Our main finding confirmed our hypothesis, indicating no significant correlation between static and dynamic balance performance based on the same CoP-related parameters. The interpretation of evidence on the relationship between static and dynamic balance is complicated [3, 7, 8, 30], likely due to the complexity of control mechanisms influencing balance performance and the wide range of balance tasks used for this purpose. Indeed, answering this question strengthened the need for separate testing procedures or training protocols (i.e., static and dynamic) in future balance research.

Similar to the results of the current study, previous literature [8, 18] demonstrated a low or no correlation between static and dynamic balance measures in a healthy population. In detail, Muehlbauer et al. [18] found no correlation between bipedal quiet stance and proactive or reactive balance in a group of older adults. Hrysomallis and colleagues [8] highlighted low correlations between a static single-leg balance task and a dynamic balance task in professional footballers.

Unlike previous literature, the strength of our study was in considering the same CoP-related parameters (i.e., Area95 and MeanVelocity) across both static and dynamic balance conditions. The lack of a clear correlation may be partly due to the fact that, while static and dynamic balance control are both regulated by the same neural structures (i.e., the cerebral cortex, basal ganglia, cerebellum, brainstem, and spinal cord), these structures are thought to contribute in different ways to the two balance conditions [31]. In static assessment, the forces that challenge balance and the demands placed on the postural control systems are minimal; unless one of these systems has been impaired due to injury or illness, the task is generally straightforward [7]. Indeed, the human bipedal stance is modelled as a single inverted pendulum where the center of mass projects in front of the ankle, creating around the ankle joint a dorsiflexor moment continuously counterbalanced by a plantar-flexor moment through the actions of postural muscles [32]. Automatic subcortical processes are thought to play a major role in controlling this anterior-posterior oscillation, and individuals are generally unaware of the minimal CoP displacements regulated by tonic contractions [31]. As a result, static postural regulation is believed to primarily occur at the brainstem and spinal levels, with neural circuits possibly modulated by local feedback loops or self-organized mechanisms, due to the stable and predictable nature of the environment [33].

Conversely, dynamic balance tasks present a greater challenge to the postural control systems. In the case of the current assessment, the unstable board task involved higher destabilizing forces, which needed to be counteracted to minimize the loss of balance and, thus, the worsening of the performance. Indeed, when engaging in dynamic tasks, ongoing changes in the surrounding environment, external forces, and sensory demands increase the cognitive involvement in balance control to accomplish goal-directed movements [31]. As a result, it is suggested that greater reliance on supra-spinal mechanisms may be required to adjust whole-body movement to the constantly changing environmental challenges [33].

### Relationship between dynamic balance and muscle strength

The second aim of the present study was to investigate whether a relationship between dynamic balance and quadriceps muscular strength exists, as a link between balance performance and muscular capacities has been previously suggested [10, 14]. Our findings did not confirm the hypothesis with the linear regression model accounting for only a small part of the variance, and only one statistically significant predictor related to strength and power. These results are in line with the available literature on the relationship between dynamic balance and isometric strength or power in healthy young adults [34, 35]. In detail, McCurdy and colleagues reported no significant correlations between single-limb standing on a wobble board and maximum unilateral squat performance in young, healthy men and women [35]. Granacher and colleagues confirmed these results while exploring the relationship between gait disruptions in treadmill walking and maximal isometric strength on the leg extension in younger and older individuals [34]. Therefore, our results support the independence of muscular and balance measures from each other when relating dynamic balance performance with maximal strength under steady and ballistic maximal contractions. As already hypothesized [14], muscle power should be more important than muscle strength in dynamic postural conditions since the initiation of any unbalance must be counterbalanced as fast as possible through opposing forces to avoid falling. Based on previous research [14, 36] a relationship between leg extensors strength and power and CoP-related parameters in a dynamic balance task could have been expected. Nonetheless, our results did not completely support our hypothesis since the model based on RFDs, FMAX_ST_ and FMAX_BAL_ account for less than 20% of the dynamic balance performance with only a significant predictor (i.e., RFD_100 − 150_). A possible explanation could lie in the role of neuromuscular efficiency during this specific time window [37, 38]. Indeed, in the 100–150 ms interval, the muscle begins to generate active force with increasing involvement of the contractile and tendon components, and the ability to recruit motor units efficiently becomes crucial for both force production and balance control [38, 39]. This time window may reflect a phase where both neuromuscular coordination and the capacity for rapid, controlled force generation contribute to the maintenance of balance, particularly in dynamic conditions where quick adjustments are required [40]. In dynamic balance tasks, the ability to generate force rapidly and accurately could support the ability to make corrective postural adjustments in response to perturbations or shifts in body position [39, 41]. However, given that the model explained low percentage of variance (i.e., 13.6% and 18.8% considering Area95 and Mean Velocity, respectively), it is important to interpret this finding with caution. The relatively low coefficient of determination suggests that dynamic balance control is a complex interplay among different anatomical structures, systems, and regulation mechanisms. Moreover, the difference in the type of neuromuscular control needed in the two motor tasks (i.e., strength production and balance on the unstable board) should be also considered. While testing of leg extensor RFD required only voluntary muscle activation with the subject seated, balance testing on the unstable board also involved modulation of muscle activation under reactive conditions with the subject in a standing position. In addition, movements to achieve balance performance during the unstable board task are more complex than pure quadriceps isometric force expression on a leg extension.

Thus, from our results, dynamic balance performance on the unstable board did not directly depend on muscular capacities (i.e., strength and power) as previous research showed in one-leg standing, gait, chair rise, timed up-and-go, and stair climbing [17]. As already explained, in dynamic balance tasks, multiple factors could affect performance due to the complexity of the motor task and the central sets involved. Therefore, the theoretical basis to predict dynamic balance improvement through muscular strength training alone [14, 42] may not be valid, at least in healthy adults.

The present study has some potential limitations that need to be acknowledged. First, all assessments were performed in a single session, which prevents evaluation of intra-individual variability but limits to assess the test–retest reliability. Second, although the sample included both males and females, potential gender differences were not analyzed due to the small sample size, which limited our ability to conduct a robust gender-stratified analysis without compromising statistical power. Third, the study focused only on quadriceps strength, whereas the contribution of other muscle groups - as ankle dorsiflexors and plantar flexors - was not assessed, potentially limiting the comprehensiveness of the analysis. Additionally, the sample consisted exclusively of healthy young adults, which may limit the generalizability of the findings to other populations.

## Conclusions

In conclusion, this study supports the lack of correlation between static and dynamic balance performance in healthy adults considering in static and dynamic conditions the same center-of-pressure parameters. This finding indicates that individuals who perform well in static balance tasks may not necessarily demonstrate comparable performance in dynamic conditions, and vice versa. Clinically, this suggests that relying solely on static balance assessments may lead to an incomplete evaluation of an individual’s postural control. Therefore, from a practical standpoint, both static and dynamic balance tests should be incorporated into clinical assessments for a more comprehensive profile of balance function. Since several neuromuscular factors may be involved in dynamic balance control, future research should also explore the neuromuscular and sensorimotor mechanisms underlying dynamic balance and its possible relationship with static balance in neurological populations.

## Electronic supplementary material

Below is the link to the electronic supplementary material.

Supplementary Material 1

## Data Availability

No datasets were generated or analysed during the current study.

## Abbreviations

95% CI: 95% confidence interval
Area95: 95th percentile ellipse area
BAL: Ballistic condition
CoP: Centre of Pressure
CV: Coefficient of variation
FMAX_BAL_: Maximal isometric strength in ballistic condition
FMAX_ST_: Maximal isometric strength in steady condition
ICC: Intraclass correlation coefficient
MVIC: Maximal voluntary isometric contraction
RFD: Rate of force development
SD: Standard deviation
ST: Steady condition
