# Supplementary material for: Exploring the relationship between static and dynamic balance performance through the same center-of-pressure parameters
Source: BMC Sports Sci Med Rehabil. 2025 Jul 30;17:221. doi: 10.1186/s13102-025-01251-x (PMC12309068; doi:10.1186/s13102-025-01251-x)
Supplement: Supplementary file 1 — Supplementary Material 1 [file 13102_2025_1251_MOESM1_ESM.pdf]

DIPARTIMENTO DI SCIENZE BIOMEDICHE

UNIVERSITÀ DEGLI STUDI DI PADOVA

TELEPHONE INTERVIEW FOR RECRUITEMENT

Questions for Inclusion:

1. **Have you ever participated in unstable board training before?**
    - (To ensure no previous experience with unstable board training)

Questions for Exclusion:

2. **Have you experienced any orthopedic injuries in the last 6 months?**
    - (To exclude subjects with recent orthopedic injuries)
3. **Do you have any history of neurological diseases?**
    - (To exclude subjects with neurological conditions)
4. **Do you currently have any sight, hearing, or vestibular disorders?**
    - (To exclude subjects with sensory disorders)
5. **Are you currently taking any medications that might affect your balance, perception, or overall health?**
    - (To exclude subjects who may be affected by medications that alter balance or other sensory functions)

General Questions for Consent:

5. **Are you willing to participate in this study and provide written informed consent once all procedures are explained?**
    - (To confirm willingness and understanding before enrollment)
